# Photoluminescence properties of a ScBO_3_:Cr^3+^ phosphor and its applications for broadband near-infrared LEDs†

**DOI:** 10.1039/c8ra01084f

**Published:** 2018-03-28

**Authors:** Qiyue Shao, Hao Ding, Leqi Yao, Junfeng Xu, Chao Liang, Jianqing Jiang

**Affiliations:** School of Materials Science and Engineering, Jiangsu Key Laboratory for Advanced Metallic Materials, Southeast University Nanjing 211189 PR China qiyueshao@seu.edu.cn; Jiangsu Bree Optronics Co., Ltd. Nanjing 211103 PR China; School of Materials Science and Engineering, Nanjing Forestry University Nanjing 210037 PR China

## Abstract

The rapid extension of solid state lighting technologies offers the possibility to develop broadband near-infrared (NIR) phosphor-converted LEDs (pc-LEDs) as novel NIR light sources. In this paper, a new NIR-emitting phosphor ScBO_3_:Cr^3+^ was synthesized by a high temperature solid state reaction method. Phase structure, spectroscopic properties, luminescent lifetime, quantum yield, emitter concentration influences and thermal quenching behavior of ScBO_3_:Cr^3+^, as well as its applications for NIR pc-LEDs, were systematically investigated. ScBO_3_:Cr^3+^ phosphors exhibit a broad absorption band ranging from 400 to 530 nm, which matches well with the characteristic emission of the blue LED chip. Moreover, Cr^3+^ ions occupy the Sc^3+^ sites with relatively low crystal field strength in the ScBO_3_ host, and therefore ScBO_3_:Cr^3+^ phosphors show intense broadband emission peaking at ∼800 nm upon excitation at 460 nm, originating from spin-allowed ^4^T_2_ → ^4^A_2_ transition of Cr^3+^ ions. The optimum Cr^3+^ concentration was determined to be ∼2 mol% with a quantum yield of ∼65%. A broadband NIR pc-LED prototype device was fabricated by the combination of ScBO_3_:Cr^3+^ phosphors and a blue LED chip, which showed a maximum NIR light output power of ∼26 mW and a corresponding energy conversion efficiency of ∼7%. The results indicate the great potential of ScBO_3_:Cr^3+^ phosphors for applications in broadband NIR pc-LEDs.

## Abstract 2

A new NIR-emitting phosphor ScBO_3_:Cr^3+^ was investigated and its great potential for applications in broadband NIR pc-LEDs was demonstrated.

## Graphical abstract

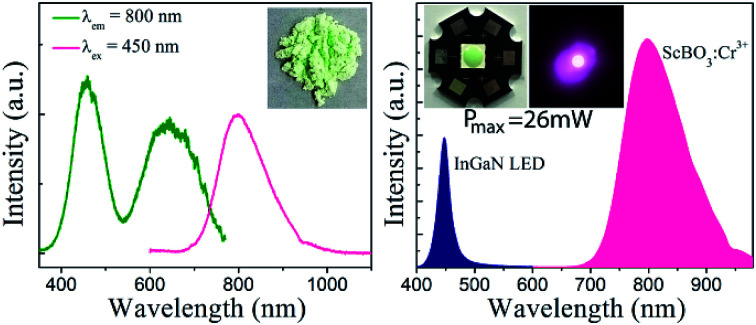

## Introduction

Since the first demonstration of high-brightness blue LEDs and subsequently white pc-LEDs,^1,2^ solid state lighting technologies have developed greatly and brought about a revolution in lighting and display industries. White LEDs are emerging as novel lighting sources to replace traditional incandescent and fluorescent lamps in numerous fields, due to their superior advantages including high luminous efficiency, environment friendliness, long lifetime, compactness and good reliability. The widely used method for producing white LED devices is the combination of a blue LED chip and certain phosphor materials with appropriate emission wavelengths. These white pc-LEDs possess simple device structure and low manufacturing cost. Besides white LEDs, the rapid developments of solid state lighting technologies also will provide a promising solution to construct broadband NIR light sources, taking advantage of the phosphor-converted LED technique.^3–7^

NIR light sources can be widely applied in a variety of fields, such as optical communication, biomedical imaging, spectroscopy, food detection, security surveillance and face (or iris) recognition.^8–12^ Incandescent bulbs, halogen lamps and AlGaAs-based LEDs are normally used as NIR light sources, however, to some extent suffering from various weaknesses. Incandescent bulbs and halogen lamps can provide continuous light emissions covering from visible to infrared ranges. However, their practical applications are limited by some drawbacks, such as large sizes, short lifetimes, low luminescent efficiency and high working temperature.^6^ In contrast, the NIR semiconductor LEDs possess small sizes, long lifetimes and high energy-conversion efficiency. Unfortunately, the LEDs normally show narrow emission bands with the largest full width at half maximum (FWHM) of ∼40 nm,^5,7^ which are inappropriate for most spectroscopic applications. A possible solution to overcome the limitations of current NIR light sources is to design the NIR-emitting pc-LEDs, which are composed of well-established InGaN blue LED chips and NIR-emitting phosphors. The NIR pc-LEDs can benefit from the outstanding merits of the blue-emitting InGaAl LED chips, such as relatively higher thermal stability, continuously increased luminous efficiency and decreased manufacturing cost. Since the invention of high-brightness blue LEDs in the mid 1990s,^1,2^ steady improvements have been achieved for InGaN-based blue LEDs. According to Haitz's law, the light output per LED package increases by a factor of twenty every decade, and the cost per lumen falls by a factor of ten.^13^ On the other hand, broadband and wavelength-tunable emissions can be expected for NIR pc-LEDs through the rational selection of emitting ions and host materials of NIR-emitting phosphors. Moreover, the continuous emission spectra will be achieved by the appropriate combination between various types of phosphor materials that possess various emission wavelengths upon excitation by the blue light.

To design NIR-emitting phosphors excited by the blue light, the emitting ions should be first of all chosen reasonably. The utilization of 4f–4f transitions of trivalent lanthanide ions is hampered by their spectrally narrow and low intensity absorption.^3,4^ For divalent or trivalent lanthanide ions (*e.g.*, Eu^2+^ and Ce^3+^) featuring 4f–5d optical transitions, it is difficult to achieve the light emissions in the NIR spectral range due to the large energy separation between 4f and 5d levels. Recently, Bi-doped crystals and glasses have attracted intense attention due to their super broadband emissions from visible to NIR.^14–16^ However, the luminescent behaviour of Bi-active centre has not been well understood, and it is still a big challenge to improve its NIR emission efficiency.^6^ Transition metal ions with incomplete 3d shells (3d^*n*^, *n* < 10) have a number of low-lying energy states, between which radiative transitions in the NIR spectral region may occur. Moreover, because they are strongly coupled to the coordination ligands in hosts, optical transitions of transition metal ions are significantly affected by the crystal field symmetry and strength. In general, transition metal ions show relatively strong and broad excitation bands, as well as wavelength-tunable broadband emissions. As typical transition metal ions, Cr^3+^ ions in the material ruby (Al_2_O_3_:Cr^3+^) have been thoroughly investigated by spectroscopists for over a hundred years.^17^ Laser-related spectroscopy of Cr^3+^ ions in a variety of crystals has also been extensively studied for the developments of tunable solid state lasers.^18^ Recently, NIR long-persistent luminescence of Cr^3+^-activated gallate phosphors has gained considerable attention due to their potential applications in optical information storage, night-vision surveillance and bio-imaging.^19–23^ Cr^3+^ ions exhibit strong absorption band in the visible spectral range and can be effectively excited by the blue light. In addition, in contrast to sharp emission lines arising from the ^2^E → ^4^A_2_ transition of Cr^3+^ ions in strong-field sites (*e.g.*, Al_2_O_3_:Cr^3+^), a tunable broad emission band arising from the ^4^T_2_ → ^4^A_2_ transition can be observed when Cr^3+^ ions occupy lattice sites with relatively weak crystal field.^17,24^ Theses optical characteristics imply that Cr^3+^ ions are an ideal choice of luminescence centres for the development of novel NIR-emitting phosphors.

In this paper, NIR-emitting ScBO_3_:Cr^3+^ phosphors were synthesized *via* solid state reaction, and their crystal structure, photoluminescence (PL) and thermal quenching properties were systemically investigated. Cr^3+^-doped ScBO_3_ single crystals have been considered as room temperature near-infrared tunable laser materials by Lai *et al.* in the mid 1980s.^25^ However, structural and spectroscopic characteristics of ScBO_3_:Cr^3+^ phosphors are far from systematic studies and especially its applicability for NIR pc-LEDs deserve further investigations. In this work, a prototype of the NIR pc-LED was also fabricated on the basis of the ScBO_3_:Cr^3+^ phosphors and a blue LED chip, and its electroluminescence properties were studied. The results demonstrate the great potential of ScBO_3_:Cr^3+^ phosphors for applications in broadband NIR-emitting pc-LEDs.

## Experimental section

### Phosphor synthesis

Sc_1−*x*_Cr_*x*_BO_3_ (*x* = 0.005–0.1) phosphors were synthesized *via* a solid state reaction method. Sc_2_O_3_ (99.99%), H_3_BO_3_ (99.95%) and Cr_2_O_3_ (99.95%) were used as starting materials. Stoichiometric amounts of raw materials were weighed and thoroughly mixed, except that 25% excess of H_3_BO_3_ was added to compensate its evaporation loss in course of high temperature sintering. Subsequently, the powder mixture was transferred into an alumina crucible and calcined in a muffle furnace at 1300 °C for 10 h. Finally, the as-prepared products were naturally cooled down to room temperature and ground to fine powders for subsequent characterization.

### Phosphor characterization

The phase structure of phosphors was identified by powder X-ray diffraction (XRD) measurements on a D8 Discover diffractometer (Bruker) with Cu Kα radiation (*λ* = 1.5406 Å). Rietveld structure refinements were conducted using the general structure analysis system (GSAS) program.^26^ The particle morphology was observed on a Sirion field-emission scanning electron microscope (FE-SEM, FEI). Diffuse reflection spectrum measurements were performed on a Cary 5000 UV-vis-NIR spectrophotometer (Varian) equipped with an internal diffuse reflectance accessory. The PL excitation spectra were measured on an F-7000 fluorescence spectrophotometer (Hitachi). The PL emission spectra were obtained on a Maya2000 portable spectrometer (Ocean Optics) using a blue LED (*λ*_em_ = 450 nm) as the excitation source. Temperature-dependent (30–250 °C) emission spectra were measured with the assistance of a self-designed heating system. The luminescent decay curves were measured by a FluoroMax-4 fluorescence spectrometer (Horiba Jobin Yvon). The quantum yield (QY) was measured by an integrating sphere equipped with a Maya2000 spectrometer and a blue LED, and the spectrometer was carefully calibrated by a standard tungsten lamp. The quantum yield is defined as the percentage of the number of emitted photons to that of absorbed photons, which can be calculated using the following equation:^27^1
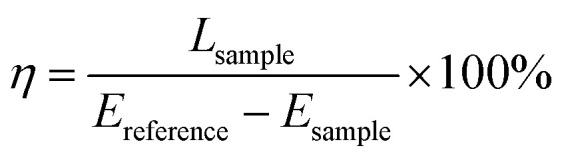
where *η* is the quantum yield, *L*_sample_ is the integrated emission intensity of the sample, whereas *E*_reference_ and *E*_sample_ represent the integrated intensities of the excitation light with and without the sample in the integrating sphere, respectively.

### NIR pc-LED fabrication and performance measurement

NIR pc-LEDs were fabricated by applying a mixture of ScBO_3_:Cr^3+^ phosphors and transparent silicon resin on an InGaN blue LED chip (*λ*_em_ = 455 nm). Photographs of the lighted pc-LED were taken by a cellphone's camera (Samsung S7), which used a Si-based photodetector covering the spectral range up to ∼1100 nm. When taking the picture, an 800 nm long-pass filter was used to remove all the visible light. For electroluminescence (EL) measurements, the packaged LED device was operated at a DC forward bias of ∼3.0 V with various injection currents, and the EL spectral distribution was recorded using an integrating sphere and a corrected Maya2000 spectrometer. The NIR light output power of the pc-LED was measured by a FieldMate light power meter (Coherent) equipped a PM10 thermopile sensor, where a 600 nm long-pass filter was used to remove the residual blue light from the LED chip. The energy conversion efficiency was defined as the ratio of the NIR light output power to the input electrical power.

## Results and discussion

### Structural analysis

**Fig. 1:**
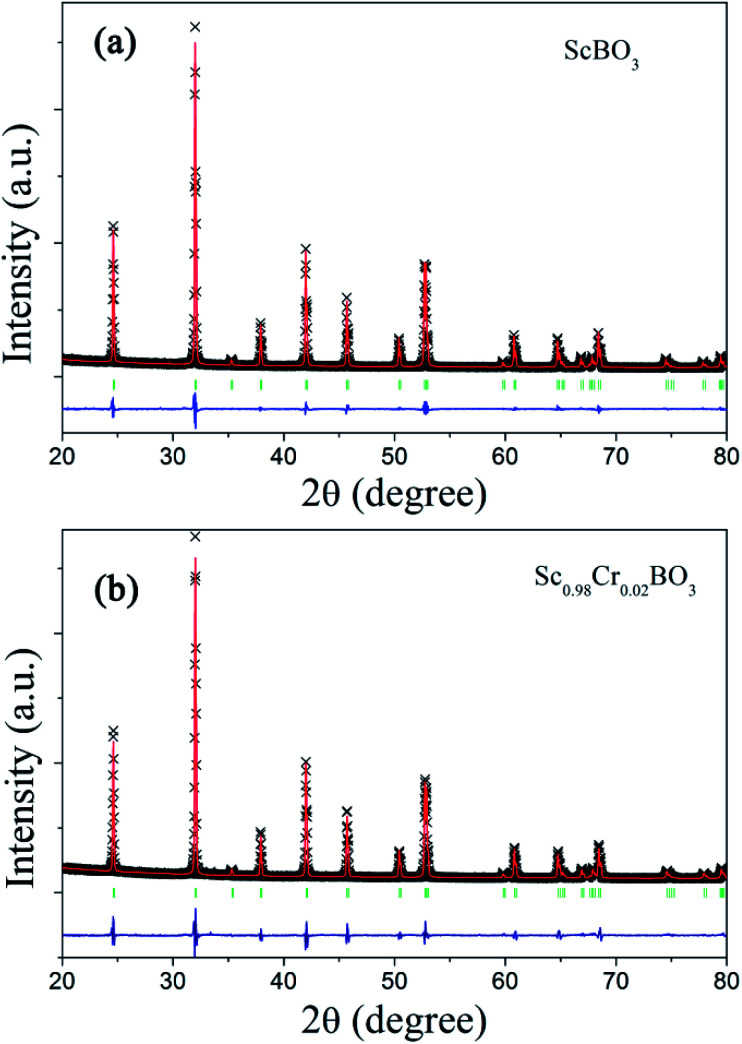
Experimental (cross), calculated (solid line), Bragg positions (tick mark) and differences (bottom) results of powder XRD refinements of (a) ScBO_3_ and (b) ScBO_3_:0.02Cr^3+^.

**Fig. 2:**
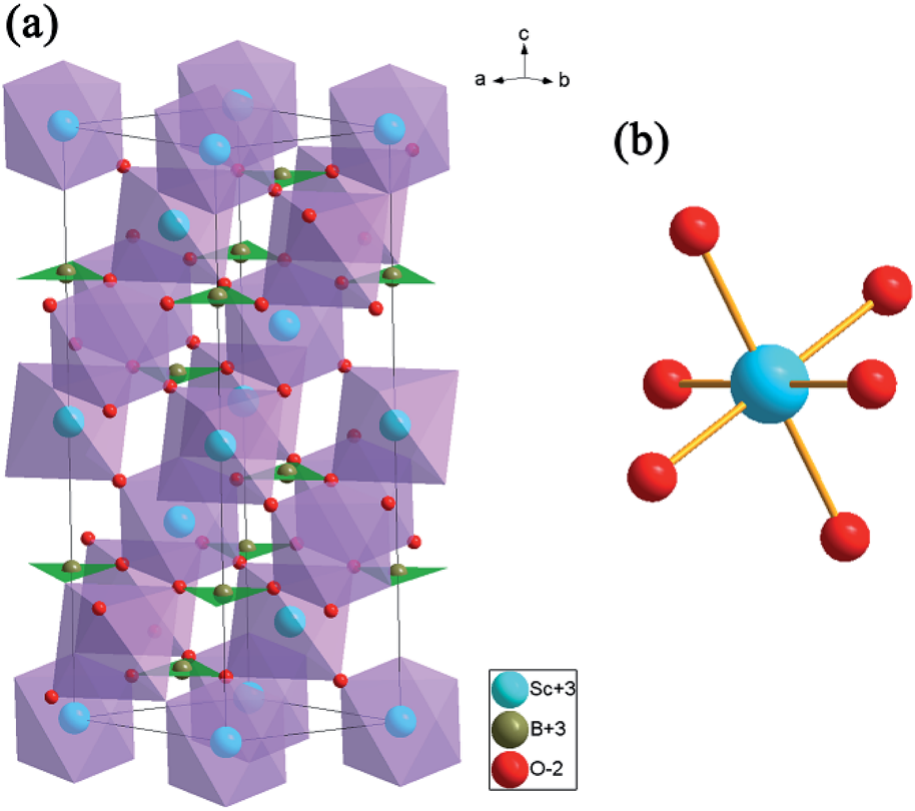
(a) The crystal structure representation of ScBO_3_ and (b) the coordination environment of [ScO_6_] octahedron.

Rietveld structure refinements of as-prepared ScBO_3_ and ScBO_3_:0.02Cr^3+^ samples were performed to identify their phase structure. The crystallographic data (ICSD# 65010) reported by Keszler *et al.* was adopted as the initial model.^28^Fig. 1 shows the refined XRD patterns of ScBO_3_ and ScBO_3_:0.02Cr^3+^, and the final refinement parameters are listed in Table 1. The refined profiles confirm the phase purity without unidentified diffraction peaks from impurity, regardless of Cr^3+^ doping. Both ScBO_3_ and ScBO_3_:Cr^3+^ are found to crystallize as a rhombohedral structure with a space group of *R*3̄*c* (no. 167). Fig. 2 displays the crystal cell of ScBO_3_ and the coordination environment of [ScO_6_] group. In ScBO_3_, Sc^3+^ ions are coordinated by six oxygen atoms and B^3+^ ions are surrounded by three oxygen atoms. ScBO_3_ exhibits a layered calcite-like structure, which is composed of [ScO_6_] octahedron and trigonal planar [BO_3_] group. Structural connectivity between the [ScO_6_] and [BO_3_] group occurs only *via* corner-sharing.

**Table 1:** Rietveld structure refinements and lattice parameters for ScBO_3_ and ScBO_3_:0.02Cr^3+^

	ScBO_3_	ScBO_3_:0.02Cr^3+^
Space group	*R*3̄*c*	
Symmetry	Rhombohedral	

**Cell parameters**		
*a* = *b* (Å)	4.7516(5)	4.7481(9)
*c* (Å)	15.2835(7)	15.2641(1)
*α* = *β*, *γ* (°)	90°, 120°	90°,120°
*Z*, volume (Å^3^)	6, 298.84(1)	6, 298.03(1)
*R*_p_ (%), *R*_wp_ (%)	6.43%, 8.57%	8.45%, 11.51%
*χ* ^2^	3.163	6.672

**Bond distance and angle**		
B–O (Å)	1.37624(1)	1.3916(18)
Sc–O (Å)	2.12198(2)	2.1124(9)
∠OBO (°)	120	120
∠OScO (°)	87.686(1), 180.000(0), 92.314(1)	87.483(25), 180.000(0), 92.517(25)

For the ScBO_3_ host, the lattice parameters are calculated to be *a* = *b* = 4.7516(5) Å, *c* = 15.2835(7) Å, *V* = 298.84(1) Å^3^, and *Z* = 6. For ScBO_3_:0.02Cr^3+^, the smaller lattice parameters are determined: *a* = *b* = 4.7481(9) Å, *c* = 15.2641(1) Å, *V* = 298.03(1) Å^3^. The effective ionic radius of Cr^3+^ is 0.615 Å when the coordination number (CN) is equal to 6, which is close to that of Sc^3+^ (0.745 Å, CN = 6) and far larger than that of B^3+^ (0.27 Å, CN = 6).^29^ Therefore, Cr^3+^ ions prefer to occupy the Sc^3+^ sites with octahedral coordination in ScBO_3_:Cr^3+^ phosphors. Due to the smaller ionic radius of Cr^3+^, the calculated cell parameters and Sc–O bond length of ScBO_3_:0.02Cr^3+^ are slightly smaller than that of pure ScBO_3_ (Table 1).

**Fig. 3:**
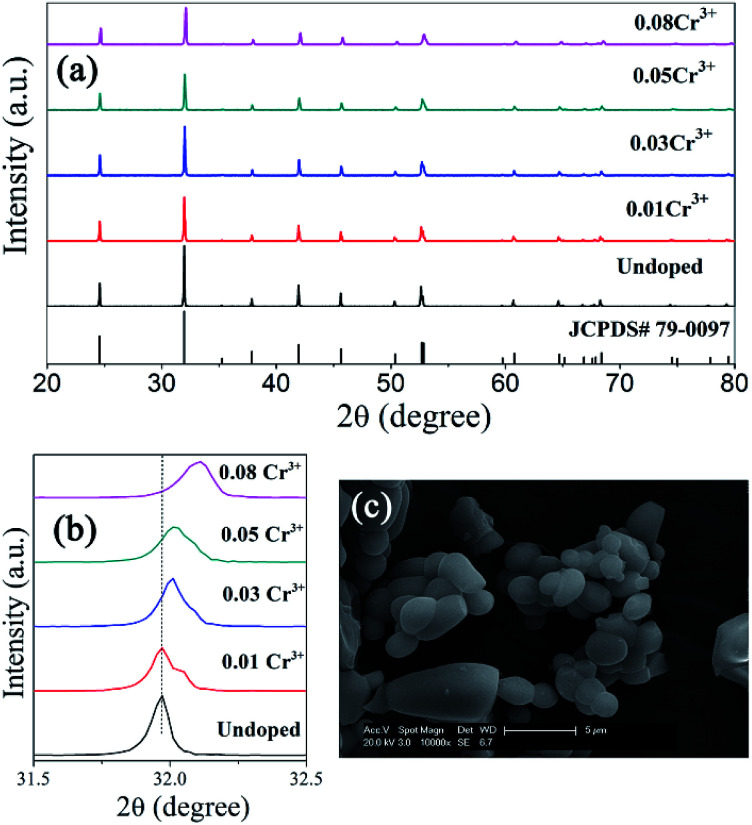
(a) XRD patters of ScBO_3_:*x*Cr^3+^ phosphors with various *x* values, together with the standard patter of a rhombohedral ScBO_3_ structure (JCPDS Card no. 79-0097) for comparison; (b) magnified XRD peak at ∼32°; (c) SEM image of ScBO_3_:0.02Cr^3+^ (scale bar: 5 μm).

XRD patterns of ScBO_3_:Cr^3+^ phosphors with various Cr^3+^ concentrations are shown in Fig. 3a. All the diffraction peaks of ScBO_3_:*x*Cr^3+^ (*x* = 0.01–0.08) phosphors agree well with those of a rhombohedral ScBO_3_ structure (JCPDS Card no. 79-0097). No impurity phase can be recognized with the increase of Cr^3+^ contents.

Noting that the XRD peaks of ScBO_3_:*x*Cr^3+^ phosphors shift to higher diffraction angle with increasing the Cr^3+^ concentration (Fig. 3b), indicative of the decrease of lattice interplanar spacing due to the smaller ionic radius of Cr^3+^. The SEM image (Fig. 3c) shows that the obtained ScBO_3_:Cr^3+^ phosphor is mainly composed of a large number of spherical particles with an average size of 1–3 μm, except that a few large-sized particles (>5 μm) are also observed.

### Spectroscopic properties

**Fig. 4:**
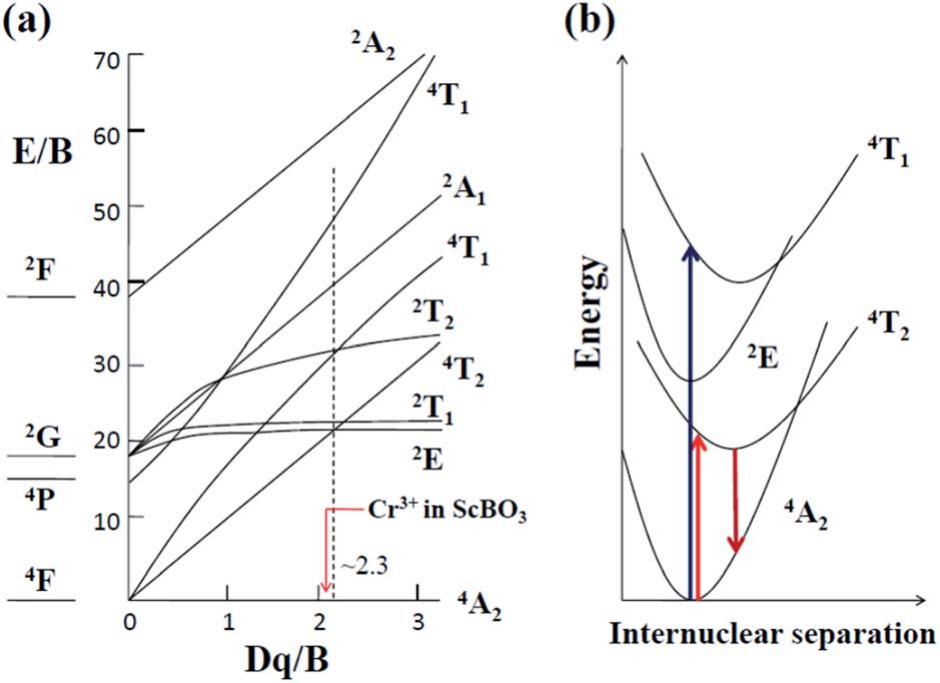
(a) Tanabe–Sugano energy level diagram for Cr^3+^ (3d^3^) ions in an octahedral crystal field (*Dq*: the octahedral crystal field parameter, *B*: Racah parameter); (b) configurational coordinate diagram of Cr^3+^ ions in a weak crystal field, *e.g.* in the ScBO_3_ host.

When Cr^3+^ (3d^3^) ions occupy lattice sites with octahedral coordination, their energy level distribution can be illustrated by Tanabe–Sugano diagram (Fig. 4a).^30^ Except for ^2^E and ^2^T_1_ levels, most of Cr^3+^ crystal field levels (*e.g.*, ^4^T_2_, ^4^T_1_, ^2^A_1_) show strong dependences on *Dq*/*B* values, where *Dq* and *B* are the crystal field strength and Racah parameters, respectively.^17,31^ Optical transitions of Cr^3+^ ions can be understood in more detail with the aid of their configuration coordinate diagrams (Fig. 4b). Noting that ^4^A_2_ and ^2^E states are derived from the t3 2 crystal field orbital, whereas ^4^T_2_ and ^4^T_1_ states are originated from the t2 2e orbital.^17,32^ Accordingly, there is a small shift in equilibrium distance between the parabolas of ^4^A_2_ and ^2^E states in configuration coordinate diagram. In contrast, the parabolas of ^4^T_2_ and ^4^T_1_ states exhibit a larger offset compared to that of the ground state ^4^A_2_. The absorption spectra of Cr^3+^-doped compounds are normally characterized by two broad absorption bands in the visible spectral range, arising from the spin-allowed ^4^A_2_ → ^4^T_2_ and ^4^A_2_ → ^4^T_1_ transitions, respectively. The emission band shapes of Cr^3+^ ions are determined by the host crystal field strength, depending on the fact whether the ^2^E or ^4^T_2_ level is lowest. As shown in Fig. 4a, ^2^E and ^4^T_2_ levels cross at *Dq*/*B* ≈ 2.3.^17^ When Cr^3+^ ions occupy high-field sites (*Dq*/*B* > 2.3), ^2^E will be the emitting state and the Cr^3+^ emission spectra is characterized by sharp emission lines (^2^E → ^4^A_2_). When Cr^3+^ ions occupy low-field sites (*Dq*/*B* < 2.3), the ^4^T_2_ → ^4^A_2_ transition will dominate the Cr^3+^ luminescence featuring a very broad emission band (Fig. 4b).

**Fig. 5:**
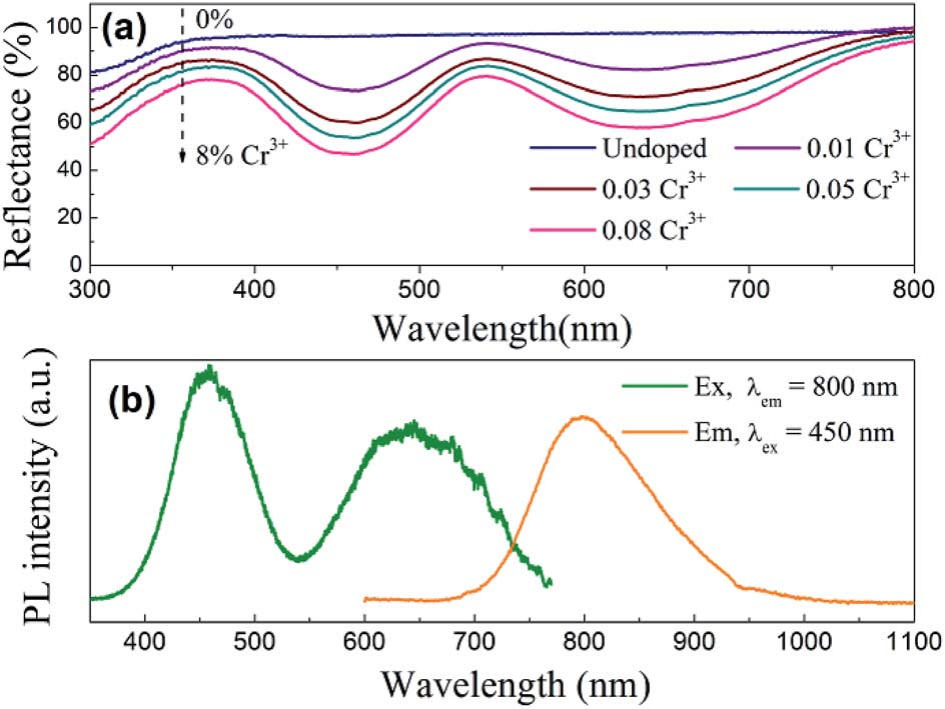
(a) Diffuse reflection spectra of ScBO_3_ and ScBO_3_:*x*Cr^3+^; (b) excitation and emission spectra of ScBO_3_:0.02Cr^3+^ phosphors.

Fig. 5a shows the diffuse reflection spectra of ScBO_3_ and ScBO_3_:*x*Cr^3+^ samples. Two broad absorption bands centred at ∼460 and 645 nm were detected after the incorporation of Cr^3+^ ions into the ScBO_3_ host, which are originated from the ^4^A_2_ → ^4^T_1_ and ^4^A_2_ → ^4^T_2_ transitions of Cr^3+^ ions (Fig. 4b), respectively. With the increase of Cr^3+^ contents, a continuous enhancement in absorptivity can be found. Fig. 5b represents the excitation and emission spectra of ScBO_3_:0.02Cr^3+^ phosphors. Upon excitation at 450 nm, the PL spectrum displays a broad emission band extending from 700 to 950 nm, with a maximum at ∼800 nm and a FWHM value of ∼120 nm. The broad emission band should be attributed to the spin-allowed ^4^T_2_ → ^4^A_2_ transition of Cr^3+^ ions (Fig. 4b). The excitation spectrum monitored at ∼800 nm is composed of two excitation bands at ∼460 and 645 nm, which is consistent with the diffuse reflection spectra of ScBO_3_:Cr^3+^ phosphors.

For pc-LED applications, the quantum yield (QY) of the phosphors is an important factor that should be considered. The quantitative excitation profiles and emission spectra of the ScBO_3_:0.02Cr^3+^ phosphor and the reference sample were recorded upon excitation at ∼450 nm using an integrating sphere (Fig. S1†). According to eqn (1), the quantum yield of the ScBO_3_:0.02Cr^3+^ phosphor is calculated to be ∼65% under 450 nm excitation. The ∼460 nm excitation band of ScBO_3_:Cr^3+^ phosphors matches well the emissions of the blue LED chips. In combination with their broadband emission at ∼800 nm, ScBO_3_:Cr^3+^ phosphors show great potential for applications in NIR pc-LED devices based on the blue LED chip.

The Stokes shift with respect to the transition between ^4^A_2_ and ^4^T_2_ levels is determined to be ∼3004 cm^−1^ by the comparison between the corresponding spectral positions of room-temperature excitation (∼15 504 cm^−1^) and emission (∼12 500 cm^−1^) bands. From the spectral positions of ^4^A_2_ → ^4^T_2_ and ^4^A_2_ → ^4^T_1_ excitation bands shown in Fig. 5b, the values of the crystal field *Dq* and Racah *B* parameters can be calculated using the following equations:^17,33^

2 *E*(^4^T_2_) = 10*Dq*

3
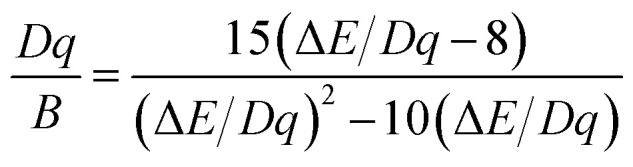
where Δ*E* is the energy separation between ^4^T_2_ and ^4^T_1_ states. The value of Δ*E* is determined by the direct comparison between the spectral positions of ^4^A_2_ → ^4^T_2_ and ^4^A_2_ → ^4^T_1_ excitation bands. The energy value (*E*) of the ^4^T_2_ level is determined from the excitation peak of the ^4^A_2_ → ^4^T_2_ transition, at the same time taking into account the influences of the Stokes shift.^31^ Herein, the difference in force constants between the ^4^A_2_ and ^4^T_2_ (or ^4^T_1_) parabolas is approximately ignored (Fig. 4b). The values of *Dq* and *B* are calculated to be ∼1400 and 651 cm^−1^, respectively. The *Dq*/*B* value is about 2.15, which is lower than 2.3, indicating that Cr^3+^ ions occupy lattice sites in ScBO_3_ with lower crystal field strength (Fig. 4a). Based on structural analysis, Cr^3+^ ions preferentially occupy the Sc^3+^ sites in ScBO_3_:Cr^3+^ phosphors. The weak crystal field should be related to longer Sc–O bond distance in ScBO_3_, because the crystal field strength is inversely proportional to the distance between the central ions and coordination ions.^31^

### Cr^3+^ concentration effects

**Fig. 6:**
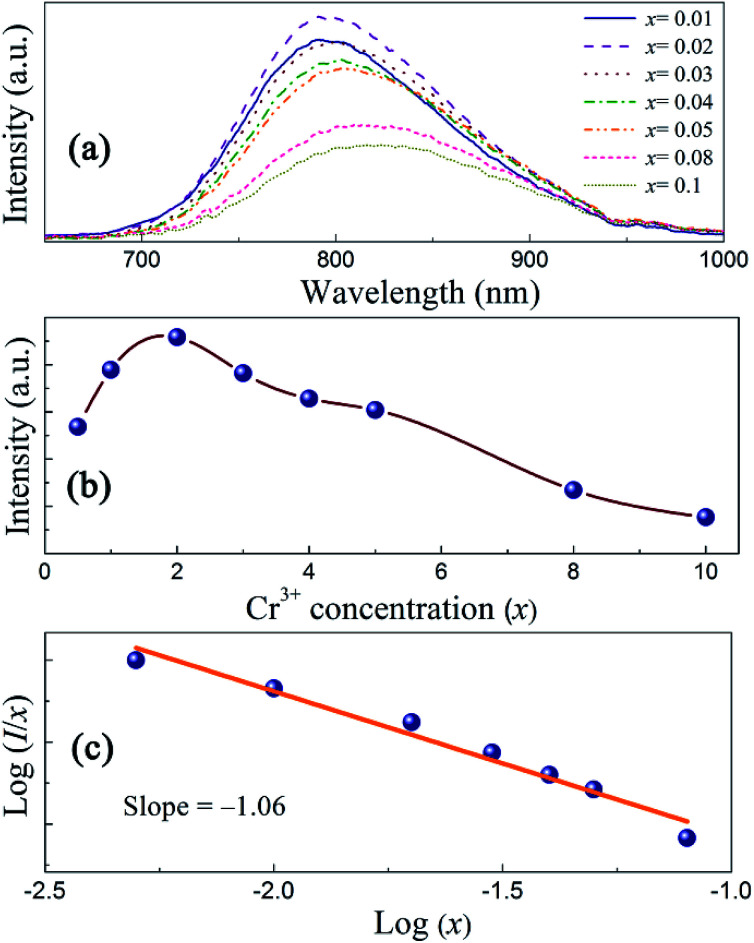
(a) Emission spectra, (b) plots of *I vs. x* and (c) log(*I*/*x*) *vs.* log(*x*) of ScBO_3_:*x*Cr^3+^ phosphors (*λ*_ex_ = 450 nm) with various Cr^3+^ contents.

Fig. 6a shows the PL emission spectra of ScBO_3_:*x*Cr^3+^ phosphors with various Cr^3+^ contents. The spectral profile changes little with the increase of Cr^3+^ concentrations. The peak wavelengths of broad emission bands of ScBO_3_:*x*Cr^3+^ phosphors exhibit a slight red-shift with increasing *x* values (Fig. S2†), despite the fact that the Sc–O distance decreases at higher Cr^3+^ contents. We believe the re-absorption between activator ions play an important role in ScBO_3_:*x*Cr^3+^ phosphor with larger *x* values. As shown in Fig. 5b, the long-wavelength excitation band of ScBO_3_:Cr^3+^ slightly overlaps with the emission band, and the re-absorption between Cr^3+^ ions will reduce the emission at the blue wing and result in the red-shift of emission band.

The emission intensities of ScBO_3_:*x*Cr^3+^ phosphors increase with increasing *x* values and reach a maximum at a critical concentration of *x* = 0.02. A further increase in the Cr^3+^ concentration results in the decrease of the emission intensity due to the concentration quenching effect (Fig. 6b). Nonradiative energy migration among luminescence centres can occur *via* exchange interaction or multipole–multipole interaction. In this case, the critical energy transfer distance (*R*_c_) can be approximately estimated by the following equation proposed by Blasse:^31^4
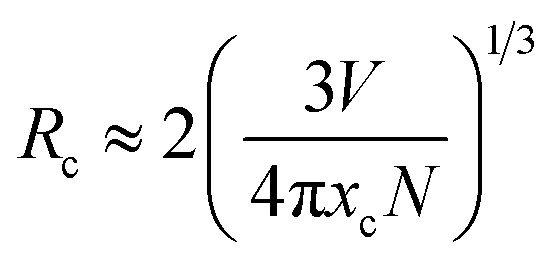
where *V* is the unit cell volume, *x*_c_ is the critical activator concentration and *N* is the number of the total activator sites per unit cell. For ScBO_3_:0.02Cr^3+^, *V* = 298.03 Å^3^, *x*_c_ = 0.02, *N* = 6, and therefore the *R*_c_ value is determined to be ∼16.8 Å. The exchange interaction is a short-range effect and usually takes effect only when the distance between activators is shorter than 5 Å. Therefore, nonradiative electric multipolar interaction is the preferred energy transfer mechanism between Cr^3+^ ions in ScBO_3_. In this case, the luminescent intensity per activator can be expressed by the following equation:^34^5
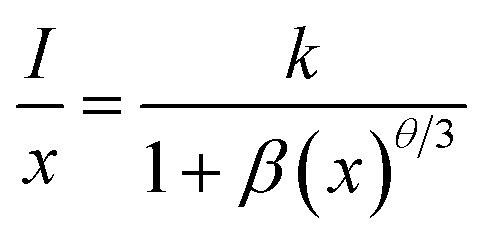
where *I* is the PL intensity, *k* and *β* are constants depending on the interaction type and the host lattice, and *x* represents the Cr^3+^ doping concentration. According to the research work of Van Uitert,^34^*θ* = 3 corresponds to the energy transfer among the nearest-neighbor ions, whereas *θ* = 6, 8, and 10 correspond to the dipole–dipole, dipole–quadrupole, and quadrupole–quadrupole interactions, respectively. As shown in Fig. 6c, the correlation between log(*I*/*x*) and log(*x*) can be fitted linearly and the slope (*θ*/3) is determined to be −1.06. The calculated *θ* value is close to 3, implying that the quenching tends to be proportional to the activator concentration and the concentration quenching mechanism of ScBO_3_:*x*Cr^3+^ phosphors can be attributed to the nonradiative energy transfer among the nearest-neighbor ions.^34–36^

**Fig. 7:**
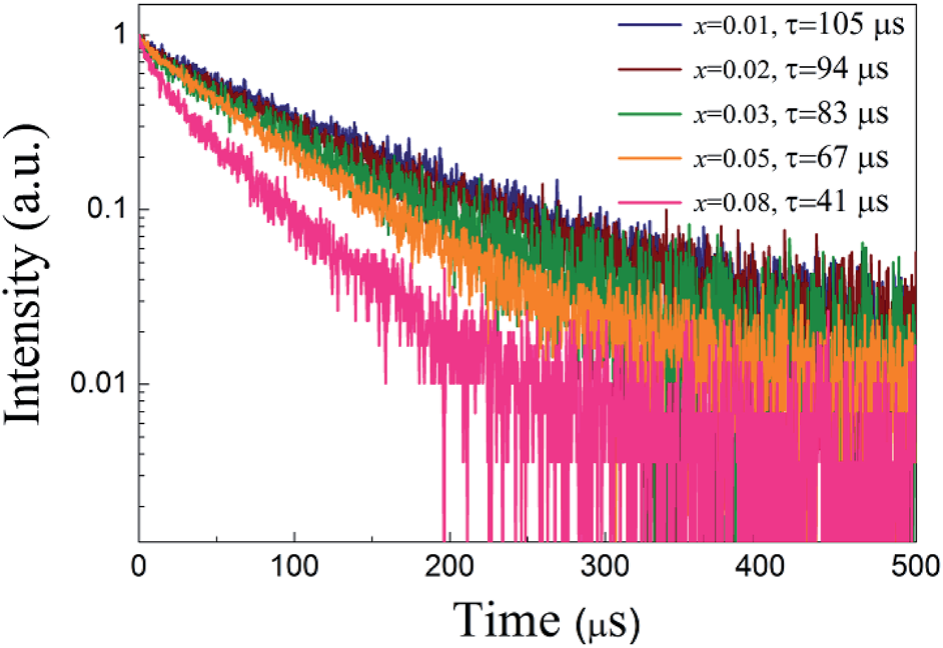
Luminescent decay curves of ScBO_3_:*x*Cr^3+^ phosphors with various Cr^3+^ concentrations (*λ*_ex_ = 450 nm).

Fig. 7 shows the luminescent decay curves of ScBO_3_:*x*Cr^3+^ phosphors with various *x* values. All the decay curves can be well fitted by a single exponential function (Fig. S3†). The luminescent lifetime (*τ*) are determined to approximately 105, 94, 83, 67 and 41 μs for ScBO_3_:*x*Cr^3+^ where *x* = 0.01, 0.02, 0.03, 0.05, 0.08. The decrease of the Cr^3+^ lifetime is attributed to the increased luminescent quenching effect at higher Cr^3+^ concentrations. The well fitting results of luminescent decay curves by a single exponential function also indicate that the Cr^3+^ ions occupy only single lattice site in the ScBO_3_ host.

### Temperature-dependent PL properties

**Fig. 8:**
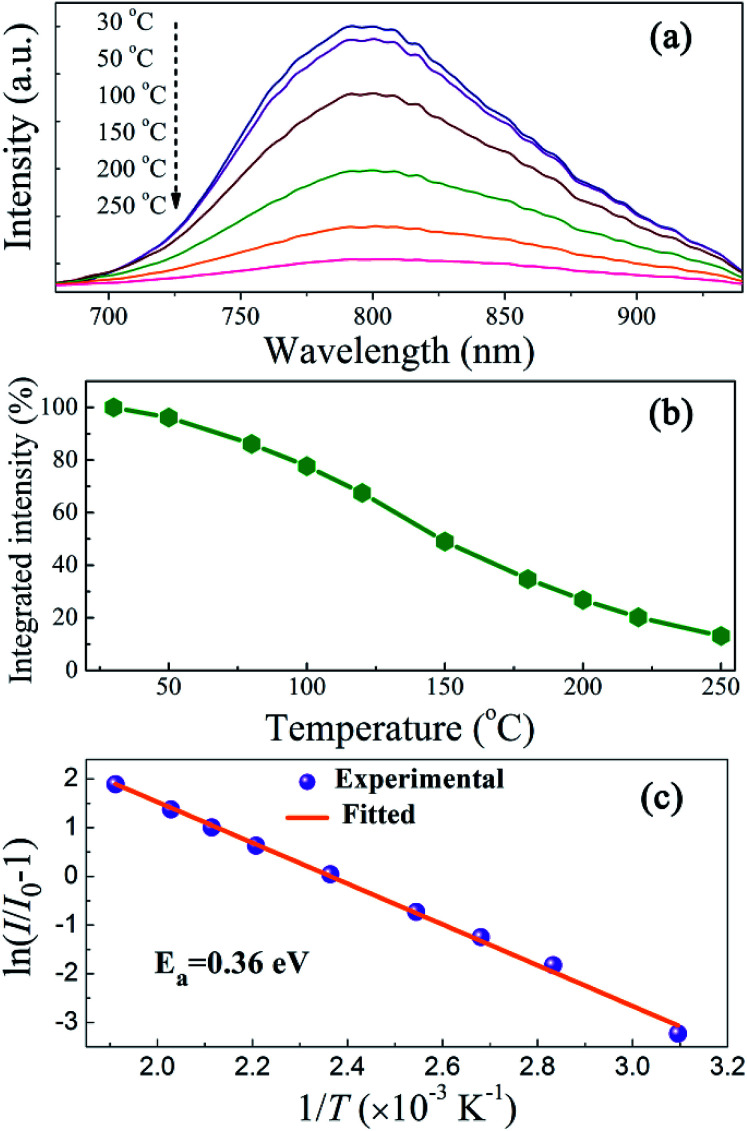
(a) Temperature-dependent emission spectra, (b) temperature-dependent normalized integrated intensities and (c) activation energy plots using the modified Arrhenius equation for ScBO_3_:0.02Cr^3+^ phosphors (*λ*_ex_ = 450 nm).

Luminescent thermal stability is another important factor of the phosphors for pc-LED applications. It is well known that the emission intensity of luminescent materials usually decreases in different degrees at elevated temperatures due to increased nonradiative transitions. The working temperature of LED devices can be higher than 100 °C,^37^ and therefore the phosphor materials should maintain their luminescent intensity at higher temperatures for pc-LED applications. Fig. 8a represents the PL spectra of ScBO_3_:0.02Cr^3+^ at various temperatures (30–250 °C), where a thermal quenching behaviour can be observed. With the temperature increasing from 30 to 150 °C, the emission intensity of ScBO_3_:0.02Cr^3+^ decreases by approximately 51% from the initial intensity (Fig. 8b). NIR-emitting ScBO_3_:Cr^3+^ phosphors show lower thermal stability than the commercial visible-emitting phosphors, such as Y_3_Al_5_O_12_:Ce^3+^ and CaAlSiN_3_:Eu^2+^, for which larger than 80% of the emission intensity at room temperature can be retained at 150 °C.^38,39^ The thermal quenching of ScBO_3_:Cr^3+^ can be caused *via* two possible routes:^31^ (1) multi-phonon emission directly across the energy gap between the excited state ^4^T_2_ and the ground state ^4^A_2_; (2) nonradiative transition through the crossing of ^4^T_2_ and ^4^A_2_ parabolas (Fig. 4b). The increased thermal quenching of ScBO_3_:Cr^3+^ is probably related to the narrow energy gap between ^4^T_2_ and ^4^A_2_ levels, as well as the larger Stokes shift of the phosphor.

The temperature-dependence of the emission intensity can be described by a modified Arrhenius equation:^40^6
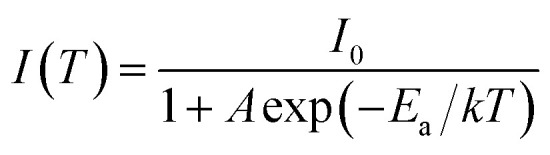
where *I*_0_ is the initial intensity, *I*(*T*) is the intensity at a given temperature, *A* is a constant, *k* is the Boltzmann constant and *E*_a_ is the activation energy for the thermal quenching. Fig. 8c depicts a plot of ln(*I*/*I*_0_ − 1) *versus* 1/*T*, and the activation energy (*E*_a_) is determined to be ∼0.36 eV for ScBO_3_: Cr^3+^ phosphors through the best fit of the Arrhenius equation.

### Fabrication and performance of NIR pc-LED

**Fig. 9:**
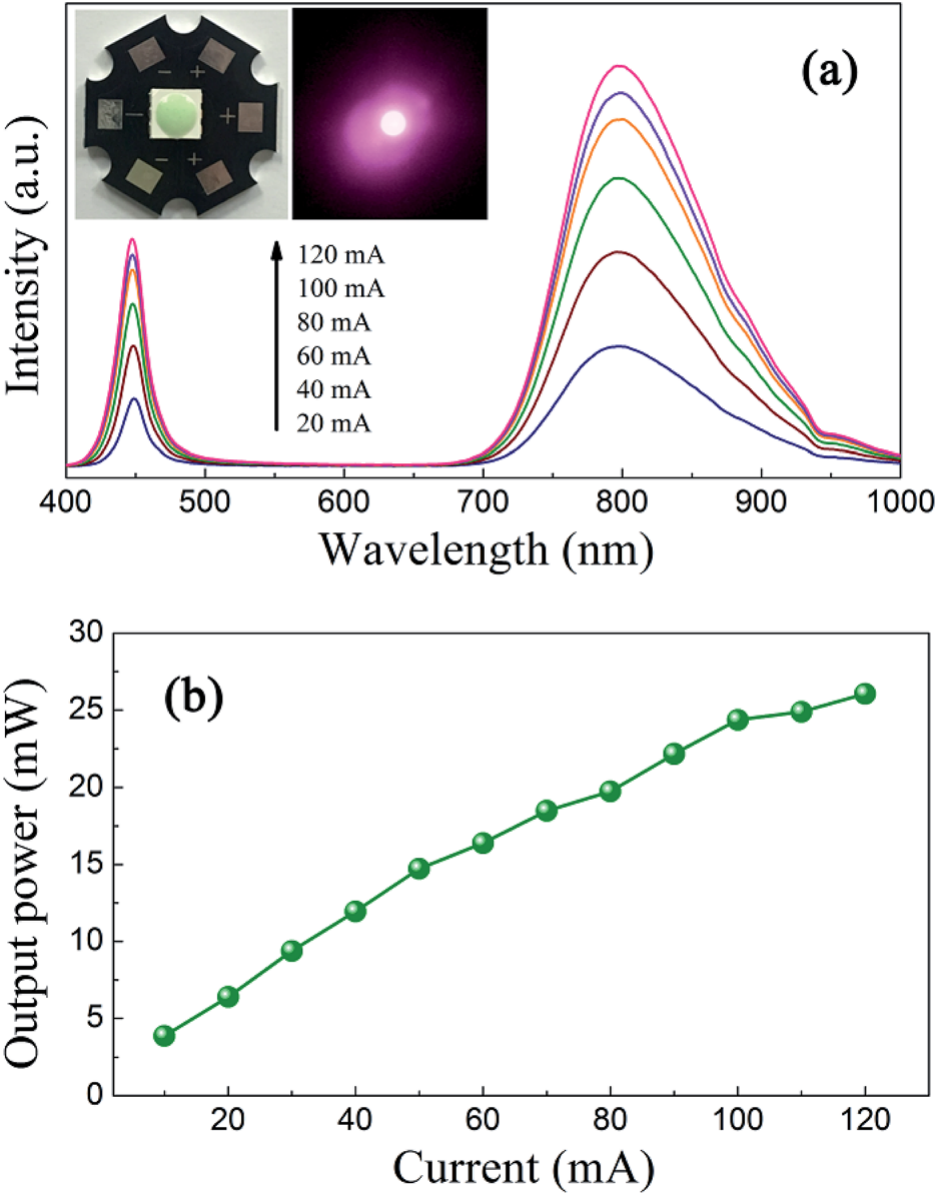
(a) Electroluminescence spectra of the pc-LED under various drive currents. The insets give photographs of the as-fabricated pc-LED and the lighted one in pseudo color. (b) The NIR light output power as a function of the drive current.

In order to demonstrate the applicability of ScBO_3_:Cr^3+^ phosphors, a NIR pc-LED prototype was fabricated by the combination of a 455 nm InGaN LED chip and the ScBO_3_:0.02Cr^3+^ phosphor. Photographs of the as-fabricated pc-LED device and the lighted one are shown in the insets of Fig. 9a. Bight NIR emission could be photographed by a cellphone's camera after the visible light was removed by an 800 nm long-pass filter. Fig. 9a shows a downshift on the emission that called here electroluminescence (EL) spectrum, even if the is no direct electron-excitation of the as-fabricated pc-LED at various drive currents. The blue emission band at ∼455 nm comes from the LED chip and the broad emission band between 700–950 nm is attributed to ScBO_3_:Cr^3+^ phosphors. With the increase in the drive current at a forward bias of ∼3 V, no obvious changes in the EL profile can be found, except for the continuous increase of the luminescence intensity.

Fig. 9b represents the dependences of the NIR light output on the injection current of the pc-LED device. A continuous increase in the NIR light output power is found with increasing the injection current. A maximum output power of ∼26 mW is achieved at a drive current of 120 mA. The energy conversion efficiency is calculated to be approximately 7%, much larger than the reported values of NIR pc-LEDs based on trivalent lanthanide doped glass phosphors.^3,4^ Noting that the energy conversion efficiency of the LED chip used here is about 20% and the blue light from the LED chip still remains for the NIR pc-LED device. The energy conversion efficiency can be further improved by engineering the package structure and utilizing more efficient blue LED chips. These results demonstrate the great potential of ScBO_3_:Cr^3+^ phosphors as an alternative NIR component for applications in broadband NIR pc-LEDs.

## Conclusions

ScBO_3_:Cr^3+^ phosphors were synthesized *via* a solid state reaction method and investigated for their structural and luminescent characteristics. The phase structure of as-prepared phosphors was confirmed by the Rietveld analysis. In the ScBO_3_ host, Cr^3+^ ions preferentially occupied Sc^3+^ sites with low-field octahedral coordination. Therefore, a broadband emission (*λ*_max_ = ∼800 nm) in the NIR spectral range between 700–950 nm was observed for ScBO_3_:Cr^3+^ upon excitation by the blue light, which was attributed to ^4^T_2_ → ^4^A_2_ transitions of Cr^3+^ ions. The luminescent quenching was proportional to the Cr^3+^ concentration in Sc_1−*x*_Cr_*x*_BO_3_ phosphors, and the maximum emission intensity was found at *x* = 0.02 with a quantum yield of ∼65%. The thermal quenching properties of ScBO_3_:Cr^3+^ phosphors were also inspected and the activation energy was determined to be ∼0.36 eV. By integrating ScBO_3_:Cr^3+^ phosphors on the blue LED chip, a broadband NIR pc-LED was obtained with a maximum NIR light output of ∼26 mW and the corresponding energy conversion efficiency of ∼7%. The results suggest that ScBO_3_:Cr^3+^ phosphors can potentially serve as conversion phosphors for broadband NIR pc-LED devices.

## Conflicts of interest

There are no conflicts to declare.

## Supplementary Material

RA-008-C8RA01084F-s001
