# Supplementary material for: Photoluminescence properties of a ScBO3:Cr3+ phosphor and its applications for broadband near-infrared LEDs
Source: RSC Adv. 2018 Mar 28;8(22):12035–42. doi: 10.1039/c8ra01084f (PMC9079304; doi:10.1039/c8ra01084f)
Supplement: RA-008-C8RA01084F-s001 [file RA-008-C8RA01084F-s001.pdf]

Electronic Supplementary Material (ESI) for RSC Advances.
This journal is © The Royal Society of Chemistry 2018

Supporting Information

Photoluminescence Properties of $ScBO_3$:Cr^{3+} Phosphor and its Applications for Broadband Near-Infrared LEDs

Qiyue Shao,*[a] Hao Ding,[a] Leqi Yao,[a] Junfeng Xu,[b] Chao Liang[b] and Jianqing Jiang[c]

[a] School of Materials Science and Engineering, Jiangsu Key Laboratory for Advanced Metallic Materials, Southeast University, Nanjing 211189, People's Republic of China

[b] Jiangsu Bree Optronics Co., Ltd., Nanjing 211103, People's Republic of China

[c] School of Materials Science and Engineering, Nanjing Forestry University, Nanjing 210037, People's Republic of China

*Corresponding author.

Email address: qiyueshao@seu.edu.cn

Tel: 86-25-52090636; Fax: 86-25-52090634

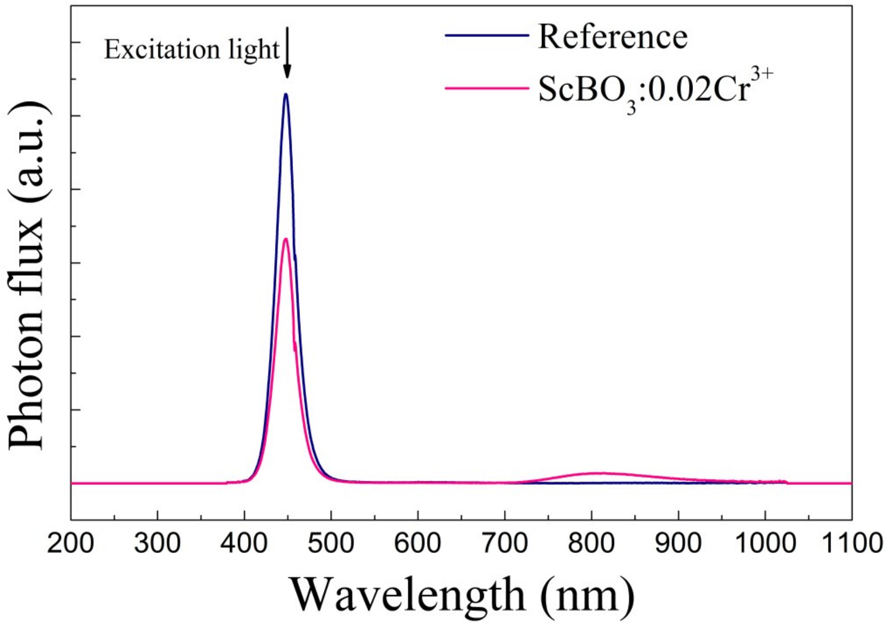

Fig. S1 The quantitative excitation profiles and emission spectra of the $ScBO_3$:0.02Cr^{3+} phosphor and the reference sample under ~450 nm excitation for quantum yield measurements.

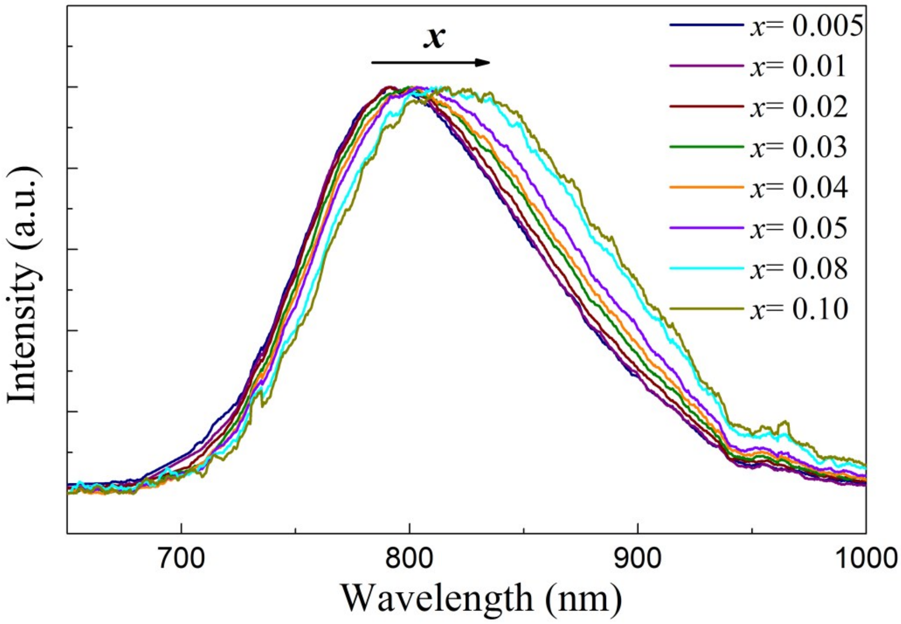

Fig. S2 Normalized emission spectra of $ScBO_3$:$x$$Cr^{3+}$ phosphors (x=0.005−0.1) upon excitation at ~450 nm.

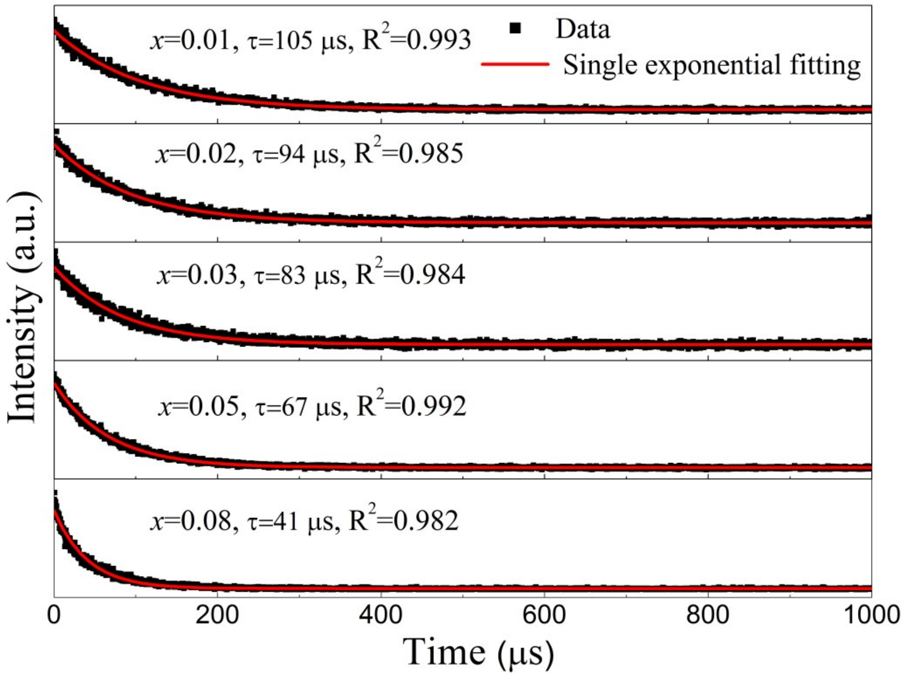

Fig. S3 Luminescent decay curves of $ScBO_3$:$x$$Cr^{3+}$ phosphors with various Cr^{3+} concentrations (λ_{ex} = 450 nm). All the decay curves can be well fitted by a single exponential function:

$$I = I_0 \exp\left(\frac{-t}{\tau}\right)$$
